# A scoping review of counselling and psychological intervention models in low income nations

**DOI:** 10.1007/s44192-026-00399-3

**Published:** 2026-03-07

**Authors:** Henok Girma, Teka Zwedie, Tigist Wuhib, Belay Tefera, Assefa Berihun, Habtamu Wondimu

**Affiliations:** School of Psychology, Addis Ababa University, Addis Ababa, Ethiopia

**Keywords:** Global mental health, Task-shifting, Low-income countries, Cultural integration, Implementation science, Scalable interventions, Treatment gap

## Abstract

There has been a persistent lack of synthesized knowledge on mental health treatment models that attune to the realities of low-income countries at large. To address this significant mental health knowledge gap, this scoping review tries to identify and synthesize the literature on psychological intervention models developed for or adapted to these contexts. After conducting a thorough search of academic databases, we went through a two-stage screening process to identify relevant articles. From those eligible studies, we collected and organized data on various theoretical and therapeutic counseling models, synthesizing the information into clear themes. The 42 included articles revealed five primary model categories: (1) Task-Shifting and Simplification, (2) Indigenous and Culturally-Grounded, (3) Systems-Level and Meta-Models, (4) Cultural Adaptation Frameworks, and (5) Technology-Based Delivery. The results indicate evidence base for the effectiveness of task-shifting simplified Western therapies and a growing movement toward validating indigenous psychologies. The findings demonstrate a clear shift toward pragmatic and culturally grounded interventions. Task-shifting is proven to be a promising approach in the field, but its effectiveness depends on having intense supervision. It is vital for research to focus on making the strategies sustainable for the long run, assessing their economic effectiveness, and comparing different models to see what works best for low-income nations.

## Introduction

Mental health is a human right that every human should have [26]. However, there is no single universally accepted definition of the term “mental health.” The concept is contested, with definitions that diverge significantly across theoretical, cultural, and political frameworks [51, 85]. For instance, the traditional medical model employs a pathogenic definition, conceptualizing mental health primarily as the absence of diagnosable mental disorders [61, 93]. In contrast, a salutogenic perspective, endorsed by organizations such as the World Health Organization (WHO and proponents of positive psychology, defines mental health as a positive state of well-being in which individuals realize their abilities, cope with normal life stresses, work productively, and contribute to their communities [22, 90]. This review adopts the broader salutogenic definition, recognizing mental health as encompassing both the absence of disorder and the presence of positive functioning, while acknowledging that this concept must be understood within cultural contexts. Cross-cultural and anthropological critiques challenge the universality of this individualistic, person-centered conceptualization [42]. From a cultural relativist perspective, Western notions of mental health that emphasize autonomy, self-actualization, and individual achievement may be incompatible with interdependent society values that prioritize interdependence, social harmony, and communal obligations. In many non-Western cultures, mental health is understood relationally, as the outcome of harmonious social relationships and fulfillment of community roles rather than individual self-realization [52, 78].

Critical psychology and socio-political frameworks, including liberation psychology and community psychology, emphasize structural determinants such as social justice, equity, and systemic oppression as central to understanding mental health [53, 80]. These theoretical perspectives reframe mental distress not as individual pathology but as an adaptive or intelligible response to adverse social conditions, including poverty, discrimination, and political violence [89].

As a result, the conceptualization of mental health spans a continuum that includes not just the absence of illness but also a holistic and politically situated state of personal and collective flourishing [4, 42, 52, 53, 93] The main ways to improve mental health and treat mental illness are through psychological interventions, which include a wide range of non-drug methods like therapy and counselling [81]. Historically, the major models of psychological intervention, such as psychodynamic, cognitive-behavioral, and humanistic therapies, emerged and were developed primarily within Western, high-income contexts [25, 50, 76]. These models are built on specific cultural assumptions about the self, emotion, and healing.

There is a substantial and persistent gap between the need for and supply of mental health services globally. This treatment gap is particularly pronounced in low-income countries (LICs), which lack both specialized mental health professionals and necessary infrastructure [48, 69]. Mental health problems are more prevalent in these settings due to higher exposure to risk factors including poverty, armed conflict, political instability, and other social determinants of health [71] (Patel and Kleinman 2003). However, evidence-based Western psychological models such as cognitive-behavioral therapy (CBT) and psychodynamic psychotherapy have been difficult to implement in low-income settings due to their resource intensity, requiring extensive training, lengthy treatment protocols, and substantial financial investment [21]. Furthermore, these imported models often fail to align with local idioms of distress, indigenous healing practices, community-based support systems, and the practical constraints faced by resource-limited health systems [44, 83].

This mismatch implies the need for evidence-based, culturally sensitive, and scalable psychological intervention models in low-income countries. [71, 83]. In response, an increasing number of studies have emerged that focus on developing innovative and locally responsive counseling approaches. [30]. These models use community health workers and non-specialists through task-shifting to address the human resources gap, but lack a consolidated evidence base.. While individual interventions are being developed and tested, there is a research gap in understanding the common features, theoretical underpinnings, and development processes of these novel approaches as a collective body of knowledge.

To address this gap, conducting a scoping review is essential as it is appropriate for mapping the extent and nature of these emerging models, identifying the key theoretical and methodological principles that guide them, and synthesizing the lessons learned from their implementation [86]. Such a review can provide a comprehensive overview of the field for researchers, policymakers, and practitioners, fostering collaboration and guiding future research and practice. Accordingly, this scoping review attempts to systematically map the existing literature on psychological intervention models designed for use in low-income countries. Guided by the research question, “What are the core elements, theoretical foundations, and development methodologies of psychological intervention models designed for low-income countries?” the scoping review aims to achieve the following objectives: first, it attempts to identify and describe the range of psychological intervention models developed or adapted specifically for use in low-income countries. Second, it analyses the core components and theoretical principles that characterize these models. Third, it synthesizes the methodologies used for the development, adaptation, and validation of these interventions in low-resource settings. And, finally, it attempts to draw implications for theoretical formulations, policy making and practical applications of counselling in low-income contexts like Ethiopia that have received little attention in previous knowledge generation.

## Method

This scoping review was conducted following the Joanna Briggs Institute (JBI) methodology for scoping reviews and adheres to the PRISMA Extension for Scoping Reviews (PRISMA-ScR) reporting guidelines. As a scoping review, this study was not registered on PROSPERO, as that database only accepts systematic reviews of health-related outcomes and explicitly excludes scoping reviews from registration.

A protocol for this scoping review followed Joanna Briggs Institute (JBI) methodology [75] as presented below.

### Eligibility criteria

The eligibility criteria were determined using the Population, theme, and Context (PCC) framework.

### Theme

The central idea involved the formulation, adaptation, or implementation of a psychological intervention or counselling model. This included therapies using pseudo-counselors or therapists with brief training, indigenous psychological frameworks, systems-level service delivery models, and strategies for cultural adaptation.

### Setting, population and contents

The context is publications in low-resource settings. The population of the study is published works related to counselling and psychological intervention models in low-income nations. The contents involved health and community systems, which included primary healthcare facilities, community-based programs, and humanitarian response efforts.

### Exclusion criteria

Studies conducted in high-income countries, those focusing on medication, and those that do not employ a specific therapeutic service delivery model were excluded from this review. Some examples include Hofmann et al. (2012). The efficacy of cognitive behavioural therapy: A review of meta-analyses. Cognitive Therapy and Research, 36(5), 427–440, was conducted in North America and Western Europe. And interventions that combine medication and therapy, which are conducted in the US Thase et al. (1997). Personality dimensions as predictors of response to cognitive behaviour therapy and pharmacotherapy for depressed outpatients. American Journal of Psychiatry, 154(10), 1546–1552.

### Search strategy

A systematic literature search was executed in several major academic databases, including PubMed, Scopus, and PsycINFO. The search was confined to articles that were published in the midst of January 2000 and August 2025 to capture the modern global mental health movement. A search string was constructed as follows: (“mental health” OR “psychotherapy” OR “counseling” OR “psychosocial”) AND (“task shifting” OR “lay health worker” OR “indigenous psychology” OR “cultural adaptation”) AND (“low income countries” OR “developing countries” OR “LMIC” OR Africa OR Asia OR “Latin America” `

### Study selection

The study selection process was designed to follow a two-phase screening protocol. The first phase was a title and abstract screening. Two assessors independently screened the titles and abstracts of the retrieved records using the eligibility criteria, and 76% of their screening found to be a match, and the agreed-upon titles were taken for further refinement. The second phase was a full-text screening. The full texts of articles filtered in the first phase were retrieved and independently assessed by the same two reviewers. Any disagreements at either phase were resolved through discussion, or, if consensus was not reached, in our case, where ten articles were in dispute, disagreements were resolved through a decision made by a third reviewer.

Data recording: A data recording form was created to extract relevant information from the included studies. Two reviewers independently filled out the charting form, and their results were compared for accuracy. The charting form contains the following variables: Author(s), Year of Publication, Country/Region of Study, Study Aim, Model Name/Type, Population Details, Study Design, and Key Findings, which are summarised in relation to the review question.

### Data synthesis

The extracted data were synthesised both numerically and thematically. A descriptive numerical summary was used to report on the characteristics of the included sources (e.g., frequency of different models, geographical distribution). A qualitative conceptual analysis was also conducted to identify and synthesise the core theoretical principles, philosophical underpinnings, and key lessons across the body of literature.

### Search and selection

Initial findings from the following databases are as follows: Google Scholar 18,300, PsycINFO 270, Scopus 1321. The search produced a total of 19,891 records. After removing duplicates and unrelated titles, a total of 1,680 unique records remained for screening the titles and abstracts. Out of these, a total of 1,595 were excluded for failing to meet the eligibility criteria. The full texts of 85 articles were then assessed for eligibility. Following this review, 43 articles were excluded for reasons such as not describing a specific therapeutic model or being based in a high-income country. Ultimately, 42 articles were selected for inclusion in this review. This process is illustrated in a PRISMA 2020 flow diagram given below.

Prisma flow chart.


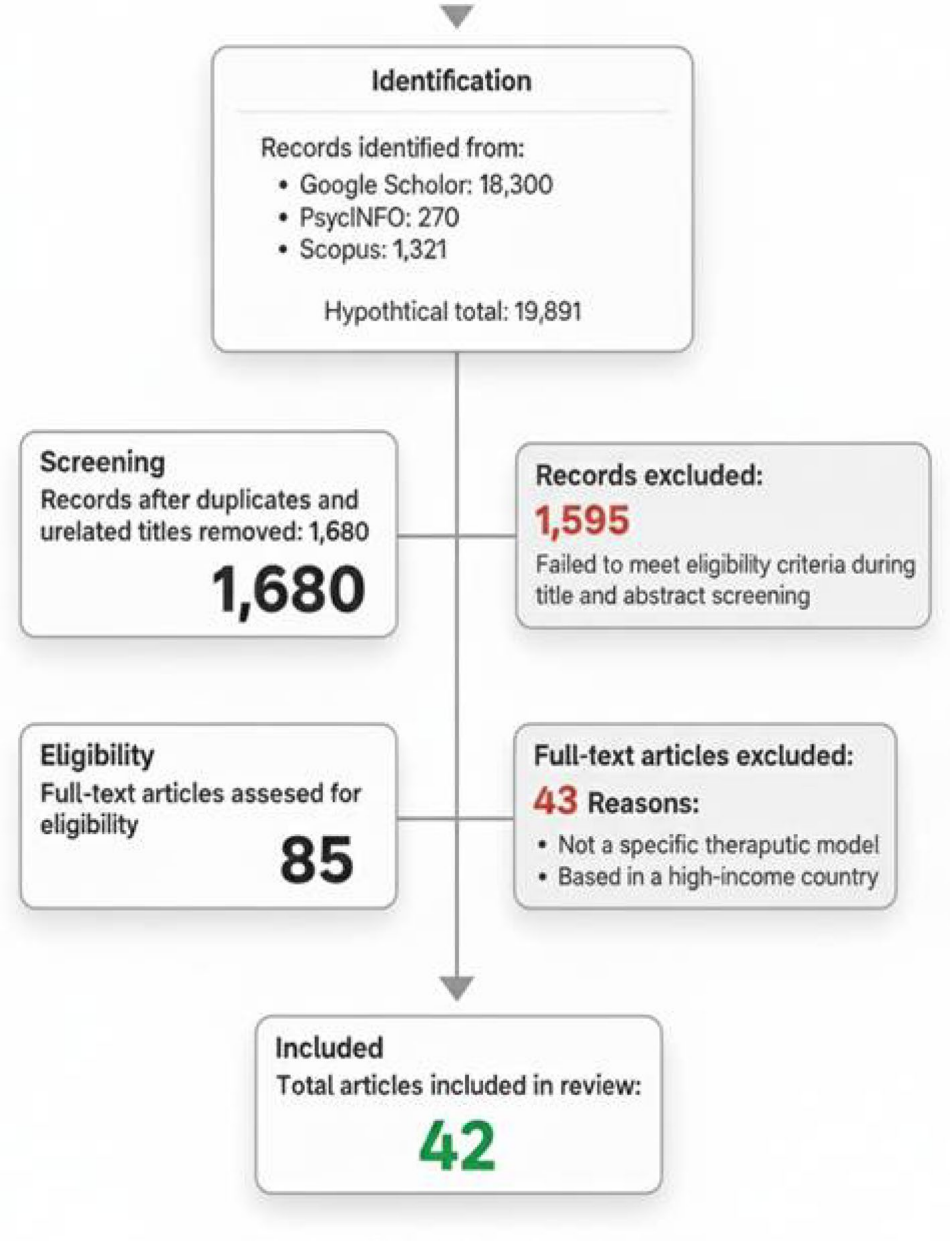


### Characteristics of included sources

The 42 studies included in this review describe models that were primarily developed and tested in sub-Saharan Africa (such as Uganda, Zimbabwe, and South Africa), with additional examples from Asia and Latin America. The literature comprises of randomized controlled trials (RCTs), qualitative studies, and theoretical papers. This involves validating specific interventions, exploring cultural concepts and proposing new frameworks.

## Results

This section is organized from a broad description of theoretical models to an in-depth analysis of their creation and testing. It begins by presenting a typology of key frameworks in global mental health, such as task-shifting, indigenous, and systems-level models, establishing what these theories are. From there, it delves into the philosophical underpinnings that motivate these approaches, exploring the why behind their development by examining drivers like pragmatism, health equity, and pluralism. Finally, the text details the practical methodology, outlining the step-by-step formulation procedures used to build the models and the rigorous validation procedures employed to confirm their effectiveness, thus explaining how these theories are constructed and proven to work.

### Typology of intervention models

A deep divide exists between methodologies focused on testing pre-specified deductive hypotheses and those that prioritize the inductive or abductive generation of theory from the ground up. The models in this review overwhelmingly favor the latter. Rather than importing and testing theories developed in high-income Western contexts, the majority of these frameworks, particularly the indigenous, culturally-grounded, and adaptation models, begin with deep qualitative exploration. Using methods like focus groups, community consultations, and textual analysis of sacred scripts, they build theory inductively. This approach directly answers the widespread dissatisfaction with the conventional practice of “corroborating” weak, decontextualized hypotheses.

#### Task-shifting and simplification models

The most widely discussed framework in the literature is task-shifting, which constitutes the majority of included studies (23 of 42 studies, 55%). This model transfers the delivery of psychological interventions from specialists to non-specialists, including nurses, clinical officers, and lay health workers [13, 18, 20, 38, 39, 46, 66, 67]. This approach simplifies complex, evidence-based psychotherapies into fundamental components, allowing them to be delivered in a structured, manualized format [21, 38, 39].

#### Indigenous and culturally-grounded models

A second category focuses on models originating from indigenous, non-Western knowledge systems (9 of 42 studies, 21%). These models are not adaptations of Western therapies but are constructed from the ground up based on local philosophical and spiritual foundations. Examples include Traditional Islamically Integrated Psychotherapy (TIIP) and Africentric counseling approaches grounded in concepts such as Ubuntu.

#### Cultural adaptation frameworks

Among the 42 included studies, 18 explicitly employed formal cultural adaptation frameworks. The Ecological Validity Model [8] was utilized in 7 studies conducted primarily in Latin American contexts, identifying eight dimensions for adaptation: language, the therapist-client relationship, metaphors, content, concepts, goals, methods, and context. The Formative Method for Adapting Psychotherapy (FMAP) [35] was employed in 5 studies, predominantly in Asian settings, outlining a five-phase, bottom-up process that emphasizes collaboration with community stakeholders. Intervention Mapping [36] was used in 6 studies, primarily for developing interventions in humanitarian and post-conflict settings in sub-Saharan Africa and Asia, combining global evidence with local qualitative needs assessments. Meta-analytic evidence supports culturally adapted interventions as more effective, particularly when targeted to specific cultural groups and delivered in clients’ native languages [28].

Several studies combined multiple approaches. Among the 18 studies employing cultural adaptation frameworks, 12 also incorporated community-based elements, including task-shifting to lay health workers or integration with traditional healing systems. Notable examples include the Friendship Bench model in Zimbabwe [1, 18, 19], which combined cultural adaptation with community-based delivery by lay counselors, and adapted Interpersonal Psychotherapy for Group (IPT-G) in Uganda [11, 88]. Only 2 studies combined cultural adaptation with technology-based delivery: the adapted telephone-delivered Common Elements Treatment Approach (t-CETA) [54] and a culturally adapted mobile mental health application piloted in South Asia.

#### Community-based and systems-level models

A third category extends from individual-focused therapy by proposing broader systems-level frameworks for service delivery. The Community-Based Rehabilitation (CBR) model is a multisectoral intervention designed to improve accessibility for individuals with psychosocial challenges, encompassing a five-component matrix: health, education, livelihood, social inclusion, and empowerment [15]. The Collaborative Stepped-Care model, tested in the MANAS trial, proposes a system in which lay counselors provide low-intensity psychosocial interventions as the initial step in primary care, with patients stepping up to pharmacological or specialist care only when necessary [20]. In humanitarian contexts, the Mental Health and Psychosocial Support (MHPSS) framework coordinates multisectoral support throughout emergency phases [64, 65]. The packages of care concept bundles evidence-based interventions for application on specific platforms within the health system [72].

### Technology-based delivery models

A smaller but essential set of articles focuses on models of service delivery that utilize technology to address barriers to access. The Behavioral Intervention Technology model provides a structured approach for designing and developing mobile health and electronic health interventions [56]. It effectively connects clinical objectives with specific technological components, features, and workflows [56]. This model is implemented in various interventions, including Computerized Cognitive Behavioral Therapy (CCBT) for treating depression [5] and telephone-delivered therapy, such as t-CETA. The latter adapts a face-to-face intervention for use by lay counselors through standard phone calls, particularly in humanitarian settings [54].

### Philosophical underpinnings

The development of scientific theories is a Multifaceted process, that can be influenced by the fundamental philosophies of researchers [45]. These philosophical paradigms that are posed and the answers that are derived [14, 31, 79]. They address necessary topics such as the essence of reality, including how we determine what is real and the role of values in research [14]. These paradigms establish a worldview that guides scientific inquiry, that influence how questions are composed and the data interpretation [29]. In psychology, three main paradigms are prominent: positivism (along with its evolution, post-positivism), constructivism, and critical realism.

A significant distinction within these paradigms is the difference between causal explanation (understanding mechanisms) and empirical prediction (forecasting outcomes), often referred to as the “two cultures” of modeling [77]. The dominant explanatory models in the field of psychology which focuses on understanding mechanisms through controlled experiments and statistical significance [92]. In contrast, predictive models prioritize accuracy in real-world situations and are increasingly being utilized in clinical applications [12]. As Fisher et al. [24] stated, confusing these two objectives can lead to theories that perform well in controlled conditions but fail to work effectively in practice, thereby increasing the gap between research and application. For example, a model that explains the cognitive mechanisms of depression may not necessarily predict treatment outcomes for diverse populations. This situation highlights the need for distinct validation strategies [9].

#### Pragmatism and evidence-based practice

A common theoretical foundation in many global mental health models is pragmatism, driven by the urgent need to address the substantial human resources crisis in low-income countries [47]. This approach emphasizes practical, scalable, and applicable methods in settings with limited resources [48]. It shows a shift away from implementing programs solely based on good intentions, toward a framework where interventions must be empirically validated.

This practical philosophy underpins the widespread adoption of task-shifting [70]. Programs such as the WHO’s mhGAP-IG and Problem Management Plus (PM +) aim to distill sophisticated therapies into essential, teachable components, enabling non-specialists and lay health workers to deliver evidence-based interventions [21, 38, 39]. This way of building the evidence base is reflected in the numerous randomized controlled trials (RCTs) that are conducted to evaluate the effectiveness of these interventions in challenging settings [19, 58, 88]. The Friendship Bench in Zimbabwe is a prime example of this approach, by utilizing a trusted group of community “grandmothers” to deliver care, with its effectiveness confirmed through rigorous trials [1], Chibanda et al. [11].

#### Indigenous knowledge and cultural congruence

Many of the articles advocate for liberating psychology by focusing on indigenous knowledge systems and cultural relevance. They criticize the uncritical use of Western psychological theories, claiming that this “psychological colonialism” is often inappropriate and ineffective in non-Western settings [62]. In particular, several models are explicitly grounded in an Africentric worldview, which emphasizes holism, spirituality, and the interdependence of the individual and the community concepts encapsulated in the term Ubuntu as the foundation for well-being [62]. This philosophy shapes counseling approaches that are rooted in African traditions of life coaching and mentorship rather than solely focusing on individual problem-solving [6]

#### Islamic psychology

Some psychological models are deeply rooted in the Islamic tradition, drawing their understanding of the human psyche and healing processes from the Qur’an and the works of classical Muslim scholars [32]. Traditional Islamically Integrated Psychotherapy (TIIP) is a framework that emphasizes the necessity of building an effective therapeutic model for Muslims on an Islamic ontological and epistemological foundation [40].

#### Pluralism and syncretism

Medical pluralism, a critical philosophy in healthcare, values and seeks collaboration with traditional healing practices, as illustrated by partnerships with South African traditional healers or Mexican Curanderismo, recognizing these systems provide valid and culturally appropriate avenues to healing. This pluralistic setting often leads to syncretism, which involves blending different explanatory models and therapeutic strategies to create a new, integrated approach. For instance, this can be seen in the combination of biomedical techniques with traditional or spiritual elements to deliver holistic patient care. [2, 16, 34].

#### Health equity and human rights

A philosophy of health equity and human rights explicitly drives many theoretical formulations. The “treatment gap” for mental health issues in low income nations is framed not merely as a resource issue but as a profound social injustice and a violation of the right to health [70]. This rights based philosophy underpins the global mental health movement and is the primary justification for large-scale initiatives designed to scale up services for the most vulnerable [70]. This concept is especially important in models designed for humanitarian and post-conflict situations. Interventions such as Narrative Exposure Therapy (NET) programs are designed to restore dignity and document human rights abuses [66, 67, 64, 65]. The Community-Based Rehabilitation (CBR) model is similarly grounded in the UN Convention on the Rights of a Person with Disabilities, viewing social inclusion and empowerment as fundamental rights.

Systems Thinking and Integration: Many recent models in mental healthcare are based on systems thinking [49]. This approach critiques fragmented, project-based interventions and advocates for integrating mental healthcare into broader health systems [91]. The philosophy suggests that sustainable and effective care necessitates strengthening the entire health system rather than just implementing isolated projects. This integration includes horizontal integration into primary care platforms [87], vertical integration across different levels of the health system through “packages of care” [49], and inter-sectoral integration with other domains such as education and social protection [37, 43]. Examples of this philosophy in action include the collaborative stepped-care model and the WHO’s strategy for integrating traditional medicine [68, 73].

### Development methodologies

#### Formulation procedures

The model construction process comprises several stages, from the collection of diverse data to performing systematic analysis, and integrating various knowledge systems into a coherent, standardized framework. Below are the steps taken to formulate and validate the models.

### Data collection

The formulation procedures primarily involved collecting qualitative data using semi-structured interviews and focus group discussions with various stakeholders, service users, caregivers, traditional healers, primary care providers, and policymakers [16, 37, 41].). For indigenous models, data were also obtained through the review of historical and sacred texts, such as the Qur’an and the writings of classical scholars relevant to Traditional Indigenous and Intercultural Psychology (TIIP) [32, 40]. In the case of adaptation models, the formulation process involved a thorough review of existing evidence-based treatment manuals to identify their essential and adaptable components [8, 37, 58].

### Data analysis

The most common method for analyzing qualitative data was thematic analysis, which was used to identify key concepts, barriers, and culturally relevant metaphors that would inform the content and structure of the model [37]. Additionally, more structured approaches were employed, such as Intervention Mapping. This six-step framework systematically transitions from needs assessment to program development [17, 66, 67] and the Formative Method for Adapting Psychotherapy (FMAP), a five-phase, community-based developmental process [41].

### Model construction

The articles discuss adapting existing evidence-based treatment approaches to better fit cultural contexts. This process included several steps, such as translating materials, adjusting metaphors to fit local contexts, and modifying therapeutic content to align with local values.s. For example, this adaptation might involve incorporating prayer or placing a greater emphasis on communal relationships [18, 58, 88].

Various approaches integrated mainstream psychological techniques and approaches within an Islamic framework [40]. While others have developed a comprehensive humanitarian response framework based on guidelines from multiple agencies like Inter-Agency Standing Committee (IASC). Other key contributors and implementers of these guidelines include the World Health Organisation (WHO), the UN Refugee Agency (UNHCR), and the UN Children’s Fund (UNICEF) [64, 65]. A crucial final step in formulating nearly all therapeutic models was manualization, which will help practitioners apply to the real world. During this stage, the principles and procedures were coded into a structured manual to guide training and ensure the fidelity of delivery [57].

### Validation procedures

Among the 42 included studies, validation approaches varied by model type and developmental stage. Thirty-one studies (74%) employed randomized controlled trials (RCTs) as their primary validation method, with 18 using cluster-randomized designs appropriate for community-level interventions. Eleven studies (26%) utilized quasi-experimental or pre-post designs, primarily for pilot feasibility testing. All studies incorporating task-shifting models (n = 23) included structured fidelity monitoring and supervision protocols to ensure intervention quality when delivered by non-specialists. Culturally adapted models (n = 18) consistently included a preliminary validation phase for assessment instruments, developing and validating locally appropriate measures before testing intervention efficacy.

### Empirical testing

The gold standard for validation is the Randomized Controlled Trial (RCT), often utilizing a cluster-randomized design for interventions at the clinic or school level. This approach has been used to evaluate the efficacy of many key task-shifted therapies [11, 18, 19, 58, 88]. Many models were initially validated through pilot studies that employed pre-post designs or quasi-experimental designs with matched control groups to assess their feasibility and effectiveness [18, 37]. A key step was validating assessment tools to ensure they were culturally and linguistically appropriate for the local context.[7, 33, 58].

#### Expert and peer review

Validation is typically an iterative process that involves consulting stakeholders and gathering feedback from the community [57]. The Formative Method for Adapting Psychotherapy involves a phase where the originally adapted manual is reviewed and revised by community stakeholders who helped develop it [41, 55]. In task-shifting models, intervention delivery is validated through expert-led supervision and fidelity monitoring, often using a structured “apprenticeship model” to maintain quality and adherence to therapy principles [57, 58].

Some models have been validated by demonstrating their practical utility. For example, the Practice-Based Evidence (PBE) approach was used to validate the TIIP model by coherently collecting and analyzing outcome data from clinical practices [40]. Frameworks have also been validated through detailed case studies that show how the models were applied in practice to achieve positive results [35]. Modeling an Afrocentric mental health counseling approach in Africa necessitates a comprehensive understanding of indigenous knowledge systems, utilizing community resources through task-shifting, employing a systematic and collaborative development process, and ensuring rigorous, context-appropriate validation.

#### Procedures followed in initiating, formulating, and validating the models

There are different procedures followed in the models developed in the literature. The procedures followed are listed below.Initiation Procedures.

Theories and models emerged as practical responses to visible crises [2], recognizing the limitations of existing psychological frameworks in low-income and non-Western contexts.

#### Catalyst

The primary catalyst for addressing mental disorders in low- and middle-income countries (LMICs) has been the substantial treatment gap, often referred to as a human resources crisis. This gap is a result of an insufficient number of specialists available to meet the population’s needs [21, 47, 70]. Acute humanitarian crises, like wars and displacements, created specific trigger for the gap, which lead to widespread psychosocial support needs that require innovative interventions [57, 64, 65]. Moreover, community traumas can prompt local leaders to seek support, exemplified by the Friendship Bench model [18]. Another significant factor has been the enlightnment of the Western models of mental health care are often inappropriate in these contexts,they can be seen as culturally incongruent, leading to client mistrust and premature dropout from treatment [32, 40, 62].

#### Foundation

Most models were developed based on initial exploratory qualitative research aimed at understanding local contexts. This research involved identifying local expressions of distress, explanatory models of illness, and community coping mechanisms, which then served as the foundation for the interventions [19, 37, 88].

Some models were initiated through a critical review of existing literature, either to gather evidence for a new approach, such as “packages of care” [74], or to create a counter-discourse to Eurocentric psychology, exemplified by African Psychology [62]. Clinical observation and practice in specific settings pointed towards the need for new theoretical approaches [40]. Additionally, systems level models were often developed through comprehensive situational analyses that mapped out existing health system resources and constraints [37, 87].

#### Formulation procedures

The model construction process comprises several stages, from the collection of diverse data to performing systematic analysis, and integrating various knowledge systems into a coherent, standardized framework.

#### Foundations in indigenous knowledge and cultural congruence

The articles indicate that effective mental health models should be established on African worldviews rather than simply adopting Western frameworks [2, 16, 60, 62, 63]. This requires a Detailed restructuring based on indigenous concepts of self, community, and wellness, rather than just making superficial changes. It’s essential to prioritize and center African worldviews [6, 16, 60, 62].

A Sincere Afrocentric approach is based on principles such as holism, which emphasizes the interconnection between the spiritual and material realms; communalism, demonstrated by concepts like Ubuntu and a spiritual understanding of the human experience [2, 3, 16, 60]. Nwoy [62] characterized this approach as a “corrective counter-discourse” to the individualism and materialism frequently dominant in the Western psychology.

#### Utilize local idioms of distress

Interventions should utilize and respect local languages and culturally relevant concepts when addressing mental health issues. For example, the developers of the Friendship Bench model centered the intervention around the Shona *term “kufungisisa*,” which means “overthinking.” This approach improved the intervention’s clarity and helped reduce stigma within the community [1, 18]. Likewise, effective research and interventions in Uganda began by specifying local mental health issues, such as *“yo’kwekyawa*,” which resembles depressive disorder [11, 88].

#### Integrate traditional practices

Instead of seeing traditional healing as a barrier, effective models should focus on collaboration and integration. This approach recognizes that many individuals seek assistance from both traditional healers and medical providers [2, 16]. Interventions can become more legitimate and effective by respectfully incorporating culturally accepted practices, such as prayer or ceremonial activities that strengthen community bonds [18, 34].

#### Systematic and collaborative formulation process

Developing new models requires a systematic and highly collaborative approach. The literature provides clear frameworks to guide this process.

#### Begin with bottom-up qualitative research

Model development should initiate with exploratory qualitative research within the target community to understand their needs, barriers, and local perspectives [37, 87].

#### Use systematic adaptation frameworks

When adapting an existing evidence-based therapy, developers should utilize systematic frameworks, such as the Formative Method for Adapting Psychotherapy (FMAP) or the Ecological Validity Model. These frameworks guide a community consultation and collaboration process to ensure that adaptations in language, metaphors, content, and goals are culturally appropriate [8, 41]. Meta-analytic evidence shows that interventions aimed at specific cultural groups are four times more effective than generic multicultural approaches [23, 82].

Manualize the Intervention. To ensure that the model can be trained, supervised, and delivered with fidelity, developers should create the final intervention in a structured, manualized format [57].

#### Holistic and integrated systems approach

Effective models acknowledge that mental health is interconnected with the broader social and health context. Therefore, interventions should be integrated into larger systems rather than functioning in isolation.

#### Integrate into primary health care

The existing primary health care (PHC) system is the most viable platform for scaling up mental healthcare. Models such as the Mental Health Gap Action Programme (mhGAP) and collaborative stepped-care are designed to incorporate mental health services within the routine functions of general health facilities [68, 73, 87]

#### Adopt a multisectoral perspective

Mental well-being is influenced by factors beyond the health sector. Models such as Community-Based Rehabilitation (CBR) highlight the importance of addressing livelihood, social inclusion, and empowerment in addition to health needs [15]. For example, the Friendship Bench effectively combines an income-generating activity with its peer support component [15].

#### Collaborating with traditional systems

An integrated system should acknowledge and build connections with traditional and informal care systems. To achieve this, it is essential to establish formal forums for dialogue and mutual referrals [16], World Health Organization [68].

#### Rigorous and contextually relevant validation

An Afrocentric model must be validated through rigorous methods that are culturally and contextually appropriate.

#### Developing local assessment tools

The validation of any model relies on accurate measurement. This process often requires the development and validation of new assessment tools based on local expressions of distress, as standardized Western tools may not be suitable [10, 33]

#### Conducting high-quality trials

Researchers have shown that it is feasible to conduct high-quality randomized controlled trials (RCTs) in African settings, a method that remains the gold standard for proving efficacy [1, 58, 88].

#### Integrating diverse methodologies

In addition to Randomized Controlled Trials (RCTs), Practice-Based Evidence (PBE) provides support for therapeutic models in real-world settings, as demonstrated by Traditional Islamically Integrated Psychotherapy [40]. The use of qualitative and mixed-methods research approaches is essential for assessing feasibility and understanding contextual factors [37, 54].

## Discussion

### Contextualization within previous literature

The findings align with and extend previous systematic reviews in several key areas. Consistent with the systematic review by Singla et al. (2017) on task-sharing interventions in low- and middle-income countries, we found that task-shifting models constitute the most widely studied approach (23 of 42 studies, 55%). However, our scoping review extends beyond their focus on efficacy to examine the theoretical foundations and development methodologies underlying these models.

The identification of cultural adaptation frameworks corroborates the meta-analysis by Griner and Smith [28], which demonstrated that culturally adapted interventions are more effective than non-adapted interventions. We extend this finding by systematically mapping which adaptation frameworks (Ecological Validity Model, FMAP, Intervention Mapping) are being employed in low-income settings and documenting their application patterns across different cultural contexts.

The review makes several unique contributions not captured in previous reviews. First, unlike systematic reviews focused on intervention efficacy (e.g., [69, 27]), our scoping review explicitly maps the theoretical foundations (pragmatism, indigenous knowledge systems, health equity, systems thinking) that drive model development. Second, we identified an emerging category of indigenous, ground-up models (n = 9 studies) that are not adaptations of Western therapies but are constructed from local philosophical and spiritual foundations, representing a distinct category not adequately captured in prior systematic reviews. Third, our synthesis of formulation procedures reveals a convergent methodological framework—from ethnographic exploration to manualization to RCT validation—that has become the standard for knowledge generation in this field.

The findings diverge from some previous reviews in important ways. While Patel et al.’s [71] Lancet Commission emphasized scaling up evidence-based interventions, our review reveals ongoing tension between “import and adapt” versus “indigenous development” approaches, suggesting the field has not reached consensus on the most appropriate pathway for intervention development in diverse cultural contexts.

### Key findings and implications for research, policy, and practice

#### Implications for research

Several key research gaps emerge that call for a shift from foundational efficacy studies to more complex, real-world investigations. A primary need is greater focus on implementation science, moving beyond proving interventions work to understanding how to sustain them long-term, integrate them into government health systems, and overcome political and financial barriers to scaling up. This includes addressing the gap in assessing economic effectiveness of various models, which is crucial for guiding policy and investment. There is pressing need for more comparative effectiveness research, such as head-to-head trials comparing different active interventions and delivery formats. This connects to a broader call for methodological pluralism, exploring approaches like Practice-Based Evidence (PBE) alongside RCTs to better evaluate complex interventions in natural settings. A critical limitation across all areas is the lack of long-term follow-up data (two years or more) on clinical outcomes and program sustainability.

Another set of gaps relates to deeper contextualization and innovation. The evidence base for integration models, such as indigenous and faith-integrated therapies like Traditional Islamically Integrated Psychotherapy (TIIP), remains nascent and requires rigorous evaluation. This includes addressing the epistemological challenge of how to safely and respectfully integrate traditional healing systems with formal biomedical healthcare. The need for context-specific research extends to neglected populations, as there is scarcity of studies on scalable psychosocial interventions for people with severe mental illness. A research gap exists at the intersection of technology and task-shifting. Future studies should explore how mobile technologies can support and enhance the work of lay health workers by improving their training, supervision, data collection, and fidelity monitoring.

#### Implications for policymakers

First, shift investment priorities from infrastructure alone to human capacity, providing sustained funding for training and long-term, institutionalized supervision of lay health workers and non-specialist providers. Second, formulate national policies and regulatory frameworks that formally endorse and delineate the roles of non-specialist mental health practitioners. In alignment with the World Health Organization’s Traditional Medicine Strategy, implement strategies for safe and respectful incorporation of Traditional Health Practitioners within the healthcare continuum [91]. Third, concentrate national scaling initiatives on a streamlined set of evidence-based, transdiagnostic interventions such as Problem Management Plus (PM +) or Problem-Solving Therapy (PST), integrated into primary care infrastructures.

#### For practitioners and policy implementers

Adopt a “Meaning-Centred” Paradigm. Work on transition thinking (Lund Biomedical) and spiritual frameworks to one that engages with local explanatory models and new client expressions of distress. This approach facilitates a more comprehensive understanding of clients’ experiences and promotes therapeutic efficacy by integrating culturally relevant narratives and frameworks.

Establishing Equitable Partnerships. Engaging traditional and faith healers is crucial as collaborators within the healthcare system. This strategy requires a shift from one-directional training models to collaborative platforms that promote mutual learning, two-way referrals, and joint care planning. Such collaborations will honour the expertise inherent in both traditional and contemporary therapeutic modalities.

Advocating for Fidelity and Quality Assurance. To ensure quality and safety in task-shifting models, two elements are indispensable: structured, manualized interventions and thorough, multi-layered supervision. The resulting emphasis on fidelity should not be viewed as a curb on therapeutic creativity. Instead, it is the essential precondition for implementing proven, evidence-based practices on a large scale.

The main conclusion from this synthesis is that the future of mental health in low-income nations depends on integration rather than isolation. No single approach can succeed alone in the evolving world of ours. The most potent and sustainable systems will be those that integrate these elements in a synergistic way.

The shortcomings seen in previous research, such as issues with sustainability in the Indian context or challenges with adherence in the CCBT trials, can be viewed as failures of integration. These failures highlight the need to embed initiatives within permanent community and health system structures and to align clinical content with engaging technological design [5, 84]. This body of work demonstrates the convergence toward a sophisticated methodological framework for knowledge generation in global mental health. The process begins with qualitative and ethnographic research to understand local needs, cultural factors, and expressions of distress. after that, a carefully adapted manualized intervention is rigorously evaluated for its efficacy, often using randomized controlled trials (RCTs). The results from these trials are then combined through systematic reviews and meta-analyses, which help create a solid evidence base. This established method from ethnographic investigation to efficacy testing, followed by evidence synthesis has become the standard for generating knowledge that is both methodologically rigorous and relevant to the context. This framework sets a robust foundation for future advancements in the field over the next decade.

## Limitations and ethical considerations

Many challenges and limitations must be considered regarding task-shifted care. Scaling up this approach carries risks such as misdiagnosis, increased stigma, and provider burnout, which require adequate support and supervision to be effectively managed [37, 87]. Additionally, the success of any model is constrained by systemic weaknesses within the broader health system [87]. Methodologically, a significant limitation in the existing evidence base is the lack of long-term (i.e., two years or more) follow-up data on clinical outcomes and program sustainability. Many studies are also affected by sampling biases. Furthermore, a potential bias arises when an organization, such as the World Health Organization, acts as both the primary developer and evaluator of its own programs, which may influence how findings are interpreted and disseminated.

The literature covers several key lessons for developing and implementing mental health models in African contexts. One essential requirement for effectiveness is cultural congruence. Models must be rooted in local worldviews, incorporate local expressions of distress (such as *kufungisisa*), and respectfully integrate traditional practices [18, 19].

Despite the counterarguments, many articles have demonstrated that task-shifting is an effective strategy for delivering evidence-based psychological therapies across numerous trials [1, 88]. The success of this approach relies on robust, structured, and ongoing supervision, which can be effectively provided through the apprenticeship model [57].

To promote sustainability, models should be integrated into existing platforms, especially within primary health care [59, 87]. Additionally, the development process must be systematic, employing a bottom-up approach that begins qualitatively and utilizes developed adaptation frameworks in collaboration with collaborators [35, 37].

## Data Availability

No datasets were generated or analysed during the current study.

## Declarations

### Clinical trials number

Clinical trial number: not applicable.
